# Alzheimer's “Prevention” vs. “Risk Reduction”: Transcending Semantics for Clinical Practice

**DOI:** 10.3389/fneur.2018.01179

**Published:** 2019-01-21

**Authors:** John F. Hodes, Carlee I. Oakley, James H. O'Keefe, Peilin Lu, James E. Galvin, Nabeel Saif, Sonia Bellara, Aneela Rahman, Yakir Kaufman, Hollie Hristov, Tarek K. Rajji, Anne Marie Fosnacht Morgan, Smita Patel, David A. Merrill, Scott Kaiser, Josefina Meléndez-Cabrero, Juan A. Melendez, Robert Krikorian, Richard S. Isaacson

**Affiliations:** ^1^The Klingler College of Arts and Sciences, Marquette University, Milwaukee, WI, United States; ^2^Kansas City School of Medicine, University of Missouri, Kansas City, MO, United States; ^3^Saint Luke's of Kansas City Mid America Heart Institute, Kansas City, MO, United States; ^4^Department of Neurology, Zhejiang University School of Medicine, Hangzhou Shi, China; ^5^Comprehensive Center for Brain Health, Charles E. Schmidt College of Medicine, Florida Atlantic University, Boca Raton, FL, United States; ^6^Weill Cornell Medicine, NewYork-Presbyterian Hospital, New York, NY, United States; ^7^Herzog Hospital, Hebrew University, Jerusalem, Israel; ^8^Centre for Addiction and Mental Health and University of Toronto, Toronto, ON, Canada; ^9^NorthShore University HealthSystem, Evanston, IL, United States; ^10^Department of Psychiatry, University of California, Los Angeles, Los Angeles, CA, United States; ^11^Pacific Brain Health Center, Pacific Neuroscience Institute, Los Angeles, CA, United States; ^12^Department of Neurology, Weill Cornell Medicine, San Juan, Puerto Rico; ^13^Jersey Memory Assessment Service, Health and Community Services, Jersey, United Kingdom; ^14^Department of Psychiatry & Behavioral Neuroscience, University of Cincinnati College of Medicine, Cincinnati, OH, United States

**Keywords:** Alzheimer's disease prevention, dementia prevention, risk reduction, precision medicine, primary prevention, secondary prevention, tertiary prevention

## Abstract

The terms “prevention” and “risk reduction” are often used interchangeably in medicine. There is considerable debate, however, over the use of these terms in describing interventions that aim to *preserve cognitive health* and/or *delay disease progression* of Alzheimer's disease (AD) for patients seeking clinical care. Furthermore, it is important to distinguish between Alzheimer's *disease* prevention and Alzheimer's *dementia* prevention when using these terms. While prior studies have codified research-based criteria for the progressive stages of AD, there are no clear clinical consensus criteria to guide the use of these terms for physicians in practice. A clear understanding of the implications of each term will help guide clinical practice and clinical research. The authors explore the semantics and appropriate use of the terms “prevention” and “risk reduction” as they relate to AD in clinical practice.

## Introduction

The terms “prevention” and “risk reduction” often are used interchangeably in medicine when referring to clinical interventions that aim to delay or prevent the onset of a disease. The implications of each term are unique and may also imply differential therapeutic effects of a proposed intervention. There is considerable debate over the use of these terms in describing interventions that aim to *preserve cognitive health* and/or *delay disease progression* of Alzheimer's disease (AD). Further, it is important to distinguish between Alzheimer's *disease* prevention and Alzheimer's *dementia* prevention when using these terms.

There has been growing interest in “preventative” interventions, ranging from pharmacologic approaches targeting disease pathology (i.e., anti-amyloid immunotherapies) to alterations in lifestyle (e.g., physical exercise, nutrition) and treatment of co-morbid medical conditions associated with an increased risk of AD dementia. The interventions aim to provide an evidence-based strategy for patients in an effort to prolong or delay the transition from preclinical (asymptomatic) AD to more advanced stages of the disease. While the end goal may be “prevention,” the means to attain this goal may include a host of “risk reduction” interventions along with a myriad of other techniques (e.g., removal of pathology, enhancement of cognitive reserve, focus on resilience).

To date, considering there is no curative treatment for AD, the terminology surrounding pre-AD dementia interventions is especially salient. Also, while prior studies have codified research-based criteria for the progressive stages of AD (1–3), there are no clear clinical consensus criteria to guide the use of these terms *for physicians in practice*. In 2013, a panel of over 100 international experts published a letter positing that dementia, including AD, can be prevented (4). The letter served as a “call to action” for clinicians and researchers to take the necessary steps to make prevention of dementia a global public health priority; however, the term prevention was not clearly defined. The purpose of this paper is to explore the semantics and most appropriate use of the terms “prevention” and “risk reduction” as they relate to the *clinical practice* of preventing or delaying the pathophysiologic state of AD, the end stage of AD dementia, and related cognitive decline.

## Prevention vs. Risk-Reduction

Prevention, most simply defined as “the action of stopping something from happening or arising,” has been cited in scholarly articles dating as early as the nineteenth century (5). More recently, “disease prevention” has been described in *Nature* as “a procedure through which individuals, particularly those with risk factors for a disease, are treated to prevent a disease from occurring. Treatment normally begins either before signs and symptoms of the disease occur or shortly thereafter. Treatment can include patient education, lifestyle modification, and drugs” (6). According to the American College of Preventative Medicine, the goal of prevention “is to protect, promote, and maintain health and well-being and to prevent disease, disability, and death” (7). As illustrated in *Nature's* definition, prevention is a multi-step process involving “treatment” at various stages in the progression of a disease. The World Health Organization (WHO) categorizes preventative interventions as primary, secondary, or tertiary prevention. Primary prevention aims to avoid disease or its associated pathology before it occurs, whereas secondary prevention entails screening to recognize disease in its earliest stages, before symptoms occur, to slow or stop its progression. Tertiary prevention involves the treatment of disease to prevent complications and minimize disability (8). It is important to note, according to *Nature* and WHO, prevention is possible even after the onset of a disease. Prevention, then, does not necessarily entail “preventing” disease how one might typically imagine but instead involves reducing the risk of adverse outcomes, such as the loss of patient autonomy, development of comorbid illnesses, and/or death as a result of disease (8).

## Alzheimer's Disease vs. Alzheimer's Dementia

**Figure 1:**
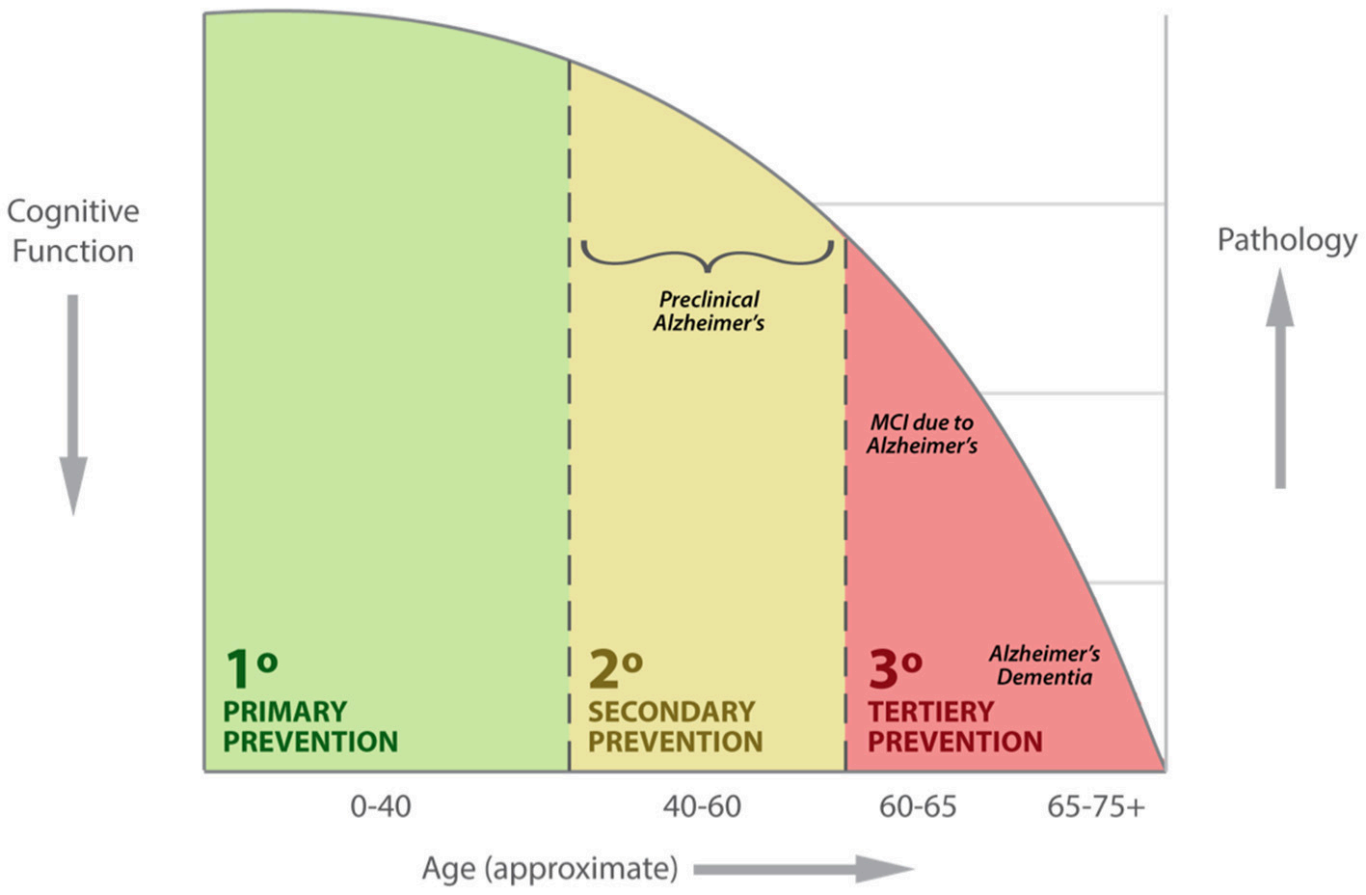
Example clinical presentation of patients including primary, secondary and tertiary prevention of AD dementia with respect to age, cognitive function and disease pathology.

A longstanding practice surrounding AD is that diagnosis begins in concert with significant memory loss and other changes in cognitive function that interfere with everyday activities. However, based on the most recent research framework criteria, AD is a progressive neurodegenerative disease that involves neuropathological changes such as the accumulation of Amyloid (A) and Tau (T) as well as Neurodegeneration (N), which begin years to decades before any cognitive symptoms of clinical AD become apparent (1). According to the model proposed by the National Institute on Aging and the Alzheimer's Association in 2011, there are three stages of AD. Stage 1 refers to preclinical AD, in which a person's brain has already started to develop specific characteristic pathologies, yet the person shows no outward signs of disease and cognitive skills remain intact (2). Stage 2 corresponds with mild cognitive impairment (MCI) due to Alzheimer's disease, in which a person has pathologic brain changes accompanied by changes in memory, language, or thinking skills that have not yet interfered with day-to-day functioning. Stage 3 is called dementia due to AD and is characterized by disease that is severe enough to impact a person's ability to complete everyday tasks independently. Recently, guidelines have been expanded to accommodate six stages, beginning with no cognitive impairment (NCI) and subjective cognitive impairment (SCI) as asymptomatic stages, mild cognitive impairment (MCI) as a prodromal stage, and mild, moderate, and severe dementia as symptomatic stages. See Figure 1 for a delineation of each of these stages concerning our framework of primary, secondary and tertiary prevention of AD dementia.

## Alzheimer's Risk Factors and Impact of Intervention

In AD dementia risk assessment, both non-modifiable and modifiable risk factors have been identified (9). Non-modifiable risk factors are traits or characteristics that are beyond personal control, including age, gender, family history, and genetics. By contrast, modifiable risk factors are those behaviors or conditions that can be changed or eliminated. The Rotterdam Study determined the seven most common modifiable risk factors involved in the development of AD dementia include smoking, midlife obesity, physical inactivity, low educational attainment, diabetes mellitus, hypertension, and major depressive disorder (10). The cumulative data in this study suggested that when these seven factors were adequately addressed, a 30% reduction in the incidence of dementia could be achieved (11). For more information, see Table 1. Other models have also suggested an even a greater degree of reduction (12, 18, 19). These estimations are likely variable due to the individual populations of subjects studied, data collection methods, and other epidemiological factors.

**Table 1:** Modifiable risk factors for AD.

**Risk factor**	**Risk of AD**	**Population attributable risk (12)**	**Number of potentially Preventable AD cases (12)**
Current Smoking	RR: 1.59 95% CI: 1.15-2.20 (13)	4.7 million AD cases worldwide may be attributable to smoking	A 25% reduction in the prevalence of smoking could potentially prevent 1 million AD cases globally
Mid-life Obesity (BMI > 30 kg/m^2^)	RR: 1.60 95% CI: 1.34-1.92 (12)	677,000 AD cases worldwide may be attributable to mid-life obesity	A 25% reduction in the prevalence of mid-life obesity could potentially prevent 166,000 AD cases globally
Physical inactivity	RR: 1.82 95% CI: 1.19- 2.78 (14)	4.3 million AD cases worldwide may be attributable to physical inactivity	A 25% reduction the prevalence of physical inactivity could potentially prevent 1 million AD cases globally
Low educational attainment	RR: 1.59 95% CI: 1.35-1.86 (15)	6.5 million AD cases worldwide may be attributable to low education	A 25% reduction in the prevalence of low educational attainment could potentially prevent 1.4 million AD cases globally
Diabetes mellitus	RR: 1.39 95% CI: 1.17-1.66 (16)	825,000 AD cases worldwide may be attributable to diabetes	A 25% reduction in the prevalence of diabetes could potentially prevent 200,000 AD cases globally
Major depressive disorder	RR: 1.90 95% CI: 1.55-2.33 (17)	3.6 million AD cases worldwide may be attributable to depression	A 25% reduction in the prevalence of depression could potentially prevent 826,000 AD cases globally
Mid-life Hypertension	OR: 1.61 95% CI: 1.16-2.24 (12)	1.7 million AD cases worldwide may be attributable to mid-life hypertension	A 25% reduction in the prevalence of mid-life hypertension could potentially prevent 400,000 AD cases globally

Recently, there has been an exponential increase in the number of studies investigating the impact of modifiable risk factor reduction on brain health, cognition, and dementia risk. Many of these demonstrate that secondary prevention strategies to reduce modifiable risk factors correlate with a decrease in the rate of progression to MCI and symptomatic AD (11, 20), suggesting “prevention,” as defined by WHO, is achievable. For example, the Finnish Geriatric Intervention Study to Prevent Cognitive Impairment and Disability (FINGER) demonstrated that the combination of a customized diet, exercise incorporating both aerobic and strength training, cognitive training, management of vascular risk factors, and social interaction over a 2-year period, improved cognitive scores among elderly people deemed to be at risk for dementia (21).

Similarly, in a systematic review of a longitudinal study, the “Lifestyle for Brain Health” (LIBRA) score was used to predict cognitive change over a period of up to 16 years. Risk variables included in this model are related to protective factors (high cognitive activity, Mediterranean diet, unsaturated fat intake, and low/moderate alcohol consumption), and predisposing factors (coronary heart disease, physical inactivity, renal dysfunction, diabetes, high cholesterol, smoking, obesity, hypertension, and depression). Researchers found a one-point progression in the LIBRA score correlated with a 9% increased risk for cognitive impairment and 19% increased risk for dementia (22). These data support the theory that “prevention” techniques, as defined by WHO, have the potential to decrease the risk of dementia significantly. Furthermore, in a recent randomized, double-blind, placebo-controlled study using a nanoparticle colloidal suspension form of curcumin with high bioavailability led to significant improvements in memory and attention in healthy adults (23). Positron emission tomography (PET) with 2-(1-{6-[(2-[fluorine-18]fluoroethyl)(methyl)amino]-2-naphthyl}-ethylidene) malononitrile (FDDNP), a chemical marker of cerebral aggregates of amyloid and tau proteins, demonstrated a substantial decrease in amyloid and tau signals in the amygdala and hypothalamic brain regions among the patients randomized to the curcumin treatment arm; decreased amyloid and tau signals were correlated with significant improvements in memory and attention abilities (23).

Other clinical trials have employed various pharmacologic interventions to delay the onset of AD. The ongoing A4 study, which is described as “a secondary prevention trial in older people with amyloid accumulation at high risk for AD dementia,” is currently exploring the efficacy of an anti-amyloid monoclonal antibody, solanezumab, in patients with positive amyloid PET scans (24). Another phase 1b study in patients with preclinical or mild AD found that 1 year of monthly intravenous infusions of aducanumab reduced brain amyloid-β (Aβ) plaques and neurofibrillary tangles in a dose- and time-dependent manner and slowed rate of cognitive decline as measured by the Clinical Dementia Rating—Sum of Boxes and Mini-Mental State Examination (25). These clinical trials, along with the lifestyle intervention studies described above, suggest that the progression and phenotypic manifestation of AD can be influenced by intervention, lending support to the plausibility of “prevention” as a goal of intervention.

Although clinical trials have demonstrated a decrease in central nervous system amyloid burden, a biomarker of AD, dementia is a complicated clinical and pathophysiological process with a diverse set of symptoms and etiologies. The Systolic Blood Pressure Intervention—Memory and Cognition in Decreased Hypertension (SPRINT-MIND) Trial examined whether treating to the lower systolic blood pressure target (120 mmHg) compared to a higher goal (140 mmHg) would reduce the risk of developing dementia and/or MCI and reduce the total volume of white matter lesions in the brain, as demonstrated by magnetic resonance imaging. Preliminary data showed a 19% lower rate of new cases of MCI and a 15% lower rate of combined MCI plus probable all-cause dementia for the intensive vs. standard treatment group (26, 27). This was the first randomized controlled trial to show that blood pressure control can delay the onset of MCI and dementia; however, it is less likely this intervention targeted AD risk in isolation, rather than simultaneously addressing the risk of vascular or other co-existing forms of dementia. While clinical trials use specific biomarkers of AD pathology, such as amyloid positivity demonstrated via amyloid PET scans, to measure the efficacy of a particular intervention, such detailed medical screening is often impractical in clinical settings.

## Terminology

One potential argument for the use of “risk reduction” as opposed to “prevention” in *general medical practice* is well illustrated through an example of a different medical condition: cancer. An individual could be a non-smoking, active, normal weight person who is conscientious about eating a healthy diet and lives in a non-toxic, safe environment with no family history of cancer, yet he or she could still develop lung cancer despite the paucity of risk factors. If a patient receives the diagnosis of a disease, it is only logical that said disease was in no way “prevented” according to the colloquial definition of the word. To prove that prevention is possible, must researchers and practitioners wait until a patient is deceased to measure the success or failure of the interventions they assigned? At what point has a disease been “prevented,” if ever? In this case, risk reduction does not equate to disease modification or prevention.

Another argument against the use of “prevention” in diseases like AD is the lack of complete knowledge about disease-specific pathology and the sequence and timing that said pathology develops and accumulates. While anti-amyloid drugs may reduce amyloid burden, it is unclear whether they can delay cognitive decline and, thus, whether they can protect against AD (28). Several studies have successfully targeted amyloid-β through the administration of drugs or supplemental interventions, including the use of bapineuzumab, solanezumab, gantenerumab, crenezumab, BAN2401, aducanumab, curcumin, and others (23, 25, 29) but have demonstrated variability in meeting primary outcome measures of a decrease in cognitive decline. Although there has been significant progress in the development of therapies, several of these interventions have not had an impact on the prevalence of AD (30).

The accumulation of amyloid-β is arguably the most commonly-accepted hypothesis for the onset of AD, but other hypotheses include the accumulation of tau, aberrant neuronal cell cycle reentry, demyelination, neuroinflammation, autophagy, metabolic dysfunction, cerebrovascular changes, and more (31). While amyloid is present in every case of AD, and is required for establishing a diagnosis, amyloid is not sufficient to cause disease and instead could represent a downstream pathological effect of other pathoetiologies (32). If there are multiple pathways in the development of AD, there may be multiple ways to treat, delay, prevent, or reduce the probability of developing AD (9). Thus, the argument can be made that before “prevention” is possible, the precise pathoetiology of AD must be better understood.

The significance of a debate over the use of “prevention” vs. “risk-reduction” is grounded in the necessity for an honest and transparent answer for patients living along the AD spectrum, as well as for their families and caregivers, particularly if these paradigms are going to be applied in clinical practice. Based solely on the example definitions of “prevention” borrowed from *Nature* or WHO, it is difficult to argue against the use of the term. However, this definition is not yet universally accepted in the medical community. Conversely, AD prevention in clinical practice may not be entirely feasible for a number of reasons beyond the direct control of the treating physician, such as patient noncompliance with prescribed interventions. Further, inconsistencies in translating AD research into clinical practice, the lack of a “one-size-fits-all” approach, and uncertainty about AD pathogenesis also complicate care. In addition, it is unclear whether the risk factors being addressed will be entirely specific to AD rather than another form of dementia. A greater understanding of the evolution of neuropathology and pathophysiology of AD may be necessary before one can definitively argue for the use of the word “prevention” in the clinical setting.

Although consensus among experts may not yet be within reach, it is worthwhile to discuss and explore these definitions. To this end, a structured clinical approach of targeted intervention may aim to delay onset (and/or reduce risk) in the area of primary AD prevention, yet for secondary and tertiary prevention, may instead seek to delay onset (and/or possibly prevent) the progression from a prodromal stage to dementia. Whether through the removal of pathological markers, enhancement of cognitive reserve, or other methods of brain resilience, further research is warranted to gain better insight into risk reduction and the feasibility of AD prevention from a practical clinical perspective. Until that time, clinicians and patients may seek ways to reduce the burden of AD and offer a proactive approach toward maintaining their cognitive health based on the mounting evidence demonstrating a direct relationship between modifiable risk factors and future dementia (33–35).

## Translating Prevention Research to Clinical Practice

Critics of the term “prevention” applied to AD may question the feasibility of preventative approaches in clinical practice, especially considering the lack of an approved pharmacological therapy and/or lifestyle approach as is traditionally utilized for most chronic disease (e.g., hypertension, hyperlipidemia, diabetes). It is important to recognize, however, that clinical research methodology is distinct from the clinical practice of Alzheimer's risk reduction. While the use of medications, supplements, or lifestyle interventions in a clinical trial might “prevent” AD according to *Nature*'s definition of the term, realistically, outcomes will vary when applied in a clinical setting due to a combination of different factors. Patient compliance, for example, plays a role in the success of prevention strategies. Non-adherence can occur in clinical practice due to a variety of reasons including stigmas and societal pressures, the high cost of medications, and an inability to perceive improvement before and after a treatment intervention (36). Another concept is the heterogeneity of treatment effect—the idea that different patients respond differently when given the same treatment for the same condition (37). Consequently, it is difficult to replicate clinical research in practice, suggesting that even if “prevention” is possible under strict guidelines, it may not be readily demonstrable in a clinical setting.

It will also be important from a clinical research perspective to study approaches geared toward optimizing the health of individuals rather than to solely diagnose and treat a disease state. It will be possible to view divergent preclinical patterns of diseases before they typically present (38). This is an important point of departure from routine medical practice within a host of disease states and facilitates a proactive, preventive approach that allows for the quantification of cognitive optimization and overall brain health, the methodologies for which have been published elsewhere (39). Emerging computation methods imposed upon serial collection of agreed upon set of biomarkers and other available lifestyle factor has promise in the AD field and already has precedent in the literature (40, 41).

## Limitations and Future Directions

“Can Alzheimer's be prevented?” is a critical question (35). Several clinical trials, such as many of those discussed above, are attempting to uncover answers through interventions intended to decrease the risk of AD. However, several obvious obstacles limit the viability of clinical research into AD prevention. One challenge is to evaluate ethical considerations. For example, the necessity of a randomized placebo-controlled study for interventions like exercise, diet, proper sleep hygiene, disease treatment, and other risk factor intervention variables would require low-risk, evidence-based risk factor modification techniques to be withheld from one group of study participants. Furthermore, most interventional studies are multimodal in nature, and therefore, it is difficult to isolate the impact of specific modifiable risk factors. While population attributable risk (PAR) provides the proportion of cases that can be ‘attributed' to a given risk factor, further studies are needed to separate the influence of individual variables contributing to increased risk for AD.

Based on the strong association between heart disease and AD, further research must explore the mechanisms by which cardiac risk factors influence the onset of dementia-related illnesses like AD. The majority of past studies have been epidemiological in nature, highlighting associations and not causation. Homologous basic science and clinical research studies are needed to quantitatively show that AD prevention is feasible and/or effective. Please see Box 1 for more information.

Box 1 **Parallels in the Field of Preventative Cardiology: A Brief Historical Overview of Terminology.**

There is significant overlap between traditional cardiovascular disease (CVD) risk factors and modifiable risk factors for AD. While the application and interpretation of “prevention” and “risk reduction” remain controversial in AD, preventative cardiology, by contrast, is a well-established field of medical practice. In fact, the preliminary draft of the Guide to the Primary Prevention of Cardiovascular Diseases, which was published in 1997 and used to supplement pre-existing guidelines for heart health put forward by the American College of Cardiology, was one of the first official sets of practice guidelines using the word “prevention” in its title (17).

In 1948, the Framingham Heart Study, a longitudinal investigation based in the greater Boston area, was launched to investigate the epidemiology of cardiac disease and identify modifiable risk factors (28). This cohort study contributed significantly to the now common knowledge that CVD and stroke can be prevented in large part through modification of lifestyle factors such as diet, weight management, exercise, and the termination of smoking, in addition to the use of medications such as aspirin, statins, and specific drugs for hypertension. Framingham also illustrated the links between coronary heart disease (CHD) and high blood pressure, low levels of high-density lipoprotein, high levels of smaller low-density lipoprotein, obesity, family history of CHD, increased serum homocysteine levels, and blood coagulation irregularities (29).

The Framingham Study proved the value of a diverse set of risk-targeted interventions in preventing cardiac events and strokes, even among those more susceptible to adverse outcomes due to a family history of heart disease. Cardiac prevention techniques are now commonly utilized by primary care providers. More recently, there has been a growing number of specialized cardiac prevention programs that focus almost exclusively on primary, secondary, and tertiary prevention. The prevention of CVD, which has been an accepted clinical practice for decades now, may serve as a model for preventative care in AD.

Globally, there are now a number of centers providing direct clinical care to patients at risk for AD dementia that, at their core, provide services aimed at risk reduction, early detection, and health promotion as components in the overall strategy of primary, secondary and tertiary prevention. In the United States, examples include the Alzheimer's Prevention Clinic at Weill Cornell Medicine and New York-Presbyterian in New York, the Alzheimer's Risk Assessment and Intervention Clinic at the University of Alabama in Birmingham, the Center for Brain Health at NorthShore University HealthSystem in Illinois, the Comprehensive Center for Brain Health Dementia Prevention Initiative at Florida Atlantic University in Florida, the Alzheimer's Prevention Clinic and Research Center in Puerto Rico (in research collaboration with Weill Cornell), and the Brain Health Center at the Pacific Neuroscience Institute, Providence Saint John's Health Center in California (39, 42–44). Several additional clinical programs are either in progress or planned across the world (e.g., Israel, China, United Kingdom). In September 2018, a symposium was convened with representatives from the majority of these centers, along with external collaborators, to find common ground among terminology, plan for ongoing collaboration and begin the process of harmonization of assessment measures and clinical research techniques.

Increased collaboration is needed to connect clinicians and researchers who are actively practicing AD risk reduction and/or prevention. This area of research is often viewed as too theoretical to study scientifically, as someone can do everything “right” and still be diagnosed with a spectrum of health problems or do everything “wrong” and live late in life with no or few significant ailments. The hope is that future clinical trials can distinguish more clearly between effective and ineffective interventions currently used to prevent, elongate the asymptomatic period, and/or decrease the risk of AD for those at all stages of the dementia spectrum.

## Conclusion

Ultimately, use of the terms “prevention” or “risk reduction” in clinical (and/or clinical research) settings remains the decision of the individual practitioner or clinician-researcher. The choice may also depend on the overall short-term and longer-term goals of the interventions. For example, even in the absence of a known modifiable risk factor, primary prevention interventions may aim to “optimize” brain health (as evidenced by improvements in objective cognitive testing) in the short-term. However, the prevention and/or risk reduction of AD may be a longer-term goal. This choice of terminology may be even more relevant when communicating with the public, as “prevention” may be a more tangible and actionable concept to grasp compared with the term “risk reduction,” which may be a source of ambiguity. Additionally, when “prevention” is defined as the end goal of intervention and reduction of risks is just one of many strategies employed to meet that goal, “risk reduction” may be too narrow a phrase to be truly interchangeable with “prevention.” Regardless, clinicians should be transparent in defining the aims of modifiable risk factor reduction techniques and avoid overpromising the expected effects for those at risk or currently on the pre-dementia spectrum of AD. For symptomatic patients, considering the current state of evidence, caution should be used when using a term such as “reverse” when referring to cognitive decline and/or the pathogenesis of the disease. Rather, aiming for symptomatic benefit, delaying progression or mitigating cognitive decline is more appropriate considering the nature of AD as a progressive neurodegenerative disease. A survey of attitudes toward these terms in a diverse group of healthcare providers and the public at large may yield valuable insights to help inform and even improve messaging further.

Overall, a greater understanding of the implications and boundaries of each term has the potential to stimulate dialogue between clinicians, clinician researchers, and the lay public. In doing so, it may facilitate the adoption of emerging strategies within preventative neurology, which may be the only definitive way to find a “cure” for AD—via a rigorous “prevention” and/or “risk reduction” approach.

## Author Contributions

JH, CO, and RI: Conceptualization; JH, CO, JO, PL, JG, NS, SB, AR, YK, HH, TR, AF, SP, DM, SK, JM-C, JM, RK, and RI: Writing original draft, review, and editing. All authors approved of the final version of the manuscript to be published.

### Conflict of Interest Statement

RI has served as a consultant for Neurotrack. JO has ownership in CardioTabs, a company that sells supplements, including a curcumin product, to support brain health. TR has received research support from Brain Canada, Brain and Behavior Research Foundation, BrightFocus Foundation, Canada Foundation for Innovation, Canada Research Chair, Canadian Institutes of Health Research, Center for Aging and Brain Health Innovation, National Institutes of Health, Ontario Ministry of Health and Long-Term Care, Ontario Ministry of Research and Innovation, and the Weston Brain Institute. The remaining authors declare that the research was conducted in the absence of any commercial or financial relationships that could be construed as a potential conflict of interest.
